# Observation of an expert model induces a skilled movement coordination pattern in a single session of intermittent practice

**DOI:** 10.1038/s41598-019-40924-9

**Published:** 2019-03-14

**Authors:** Jason Friedman, Maria Korman

**Affiliations:** 1 Department of Physical Therapy, Stanley Steyer School of Health Professions, Sackler Faculty of Medicine, Tel Aviv University, Tel Aviv, Israel; 2 Sagol School of Neuroscience, Tel Aviv University, Tel Aviv, Israel; 3 Edmond J. Safra Brain Research Center for the Study of Learning Disabilities, University of Haifa, Haifa, Israel

## Abstract

We tested how observation of a skilled pattern of planar movements can assist in the learning of a new motor skill, which otherwise requires rigorous long-term practice to achieve fast and smooth performance. Sixty participants performed a sequence of planar hand movements on pre-test, acquisition, post-test and 24 h post-training blocks, under 1 of 4 conditions: an observation group (OG), a slowed observation group (SOG), a random motion control group (RMCG) and a double physical training control group (DPTCG). The OG and SOG observed an expert model’s right hand performing the study task intermittently throughout acquisition, RMCG observed random dots movement instead of a model. Participants in the DPTCG received extra physical practice trials instead of the visually observed trials. Kinematic analysis revealed that only in conditions with observation of an expert model there was an instant robust improvement in motor planning of the task. This step-wise improvement was not only persistent in post-training retests but was also apparently implicit and subject to further incremental improvements in movement strategy over the period of 24 hours. The rapid change in motor strategy was accompanied by a transient within-session increase in spatial error for the observation groups, but this went away by 24 h post-training. We suggest that observation of hand movements of an expert model coaligned with self-produced movements during training can significantly condense the time-course of ecologically relevant drawing/writing skill mastery.

## Introduction

Observation is one of the most powerful ways of transmitting behaviours. Humans learn many skills (procedural, “how-to” knowledge) through observation^1^. Can observation of skilled movement patterns intermittent with physical practice result in a condensed time-course of motor learning, saving the trainee multiple sessions of repetitive practice? In many fields, large amounts of physical practice are needed to achieve mastery of motor skills^2^, and thus ways to decrease the amount of learning necessary can be highly advantageous.

Skill learning is a multi-stage process requiring multiple practice sessions. Voluminous studies have investigated the behavioural, kinematic and neuroplasticity outcomes of motor skill learning following physical practice. Physical practice (on-line learning) triggers a cascade of physiological and structural changes in the brain regions engaged during the task performance as well as changes in other brain regions, processes known as “memory consolidation”^3^. On the behavioural level, these changes are expressed as between-session improvements (off-line learning) of task performance or only in stabilization of performance^4,5^. Training related factors such as the number of task repetitions, feedback and instruction are critical in determining the course of on-line and off-line skill learning^6–9^.

In learning a sequence of planar hand movements passing through several targets, e.g., in handwriting, extensive training is needed to achieve fast and smooth performance through formation of new movement elements (primitives) and concatenation of movement components^10^. In many cases of highly trained movement sequences, such as in speech production^11^, musical instrument playing^12^, and finger sequences during typing^7^, movement elements are produced with such an extensive spatial and temporal overlap that with recurrent practice a new entity (motor primitive) that is different from the sum of the elements which comprise it, is created. Motor task representation incorporating consecutive anticipated movements is known in the literature as “chunking”^10,13–15^. The behavioural correlate of chunking, whereby in a well-trained motor sequence the generation of a given motor element is influenced by the next concatenated movement, is often called “co-articulation”; the term co-articulation initially referred to articulatory overlap of speech sounds^16^, but is also used to designate the prominent overlapping of well-trained sequences of hand or finger movements^7,10,17^. Previous studies^10,18^ have shown that co-articulation of a sequence of planar hand movements in a task gradually develops through multiple training sessions and is subserved by changes in brain activity patterns^19^.

Motor memories can be induced by observing someone performing an action, even without actual movements produced by the learner^20^. The behavioural outcomes of observational learning, however, are different from  those following physical practice^21^. A combination of observational and physical training has been shown to be very effective and even superior to physical practice alone^22^. Observing others performing a motor skill has been shown to benefit acquisition and learning, and importantly can even lead to skill consolidation of the observer^21^.

Observational learning is subserved by the mirror neuron system involved in understanding others’ actions and intentions behind these actions^23^. A skilled, or expert model is someone who demonstrates proper execution of the to-be-learned skill to the trainee with little to no error^24,25^. Observation of hand movements of a skilled model was shown to enhance motor skill acquisition^26,27^. It is supposed that observers learn the expert’s motion strategy and apply it to produce appropriate coordination patterns in the novel task^22,28^. However, it is not plausible that the observed movements are transduced directly into an observer’s brain representation of actions, otherwise, an instant, accurate and long-lasting imitation of the expert’s behaviour would be found, which is not the case^29,30^. An explicit understanding of how to improve in the task may contribute to the procedural learning based on observation^31–33^.

In summary, there is evidence of observational learning of sequence information, however, it is not yet clear whether observation of skilled motor performance results in a rapid qualitative (change in the motor primitives) or only quantitative (optimization of the already existing motor primitives) change of a novel motor sequence performance. Additionally, it is not known what are the costs of the observation-based gains in performance (e.g., speed-accuracy trade-off) when observation trials partially substitute physical practice? Here,  we combined observation and physical practice during training, to avoid the differences observed when solely observation is used^34^. In ecological settings of training, it is a common situation to have intermittent practice, where actual performance is supported or guided by demonstration. If indeed there is a long-term qualitative, representational shift of the trained motor task following observation practice, what is the time-course of the acquired knowledge, specifically, is it a subject for further off-line consolidation processes? Finally, is the task representation following observation practice movement sequence (order)- or scale (length) specific?

To address these questions, in the current study we employed an ecologically valid task similar to the letter handwriting skill. The task required production of a sequence of planar trajectories passing through several targets^10^. The learning of this task requires multi-session training, across which a process of gradual replacement of straight trajectories by longer curved ones occurs, i.e. co-articulation of movement components leading to the formation of new curved, faster and smoother movement primitives^10^. Thus, a well-trained subject, an “expert” model, executes the task in a radically different way compared to a novice without compromising accuracy of performance. The acquisition of a skilled geometrical motion primitive was shown to be dependent on the affordance of visual feedback from the trainee’s hand^18^.

Here we tested in young right-handed healthy adults the hypothesis that observation of a given sequence of handwriting-like trajectories connecting four target points performed by an expert intermittent with physical practice of the same sequence will facilitate the time-course of learning relative to physical practice alone or observation of a random movement. First, we trained a naïve subject through a well-established multi-session training protocol to the level of expert^10^. The video of “expert” hand movements holding a stylus from the last (10-th) practice session was used as a model in observation trials of the novice trainees, either at the original speed of performance or in a slowed version, at a 1/3 of the original expert speed, which may allow the participants to better observe the movement. As observational learning may rely on purely perceptual (observe change in movement correlated stimuli, e.g., path, but not the movement of the effector themselves) or motor (observe effector movements without correlated stimuli/object movements) information or both^35^, we allowed only the motor-related information (hand movement) to be observed. Thus, no visualization of the path (ink trace) was afforded in any of the observation, physical training or test trials. Nevertheless, subjects may have been able to infer the path based on observation of the hand motion^36,37^.

## Results

The participants were all able to perform the task. One subject (from group 2) was removed from the analysis because of incorrectly performing the task in the first session.

**Figure 1:**
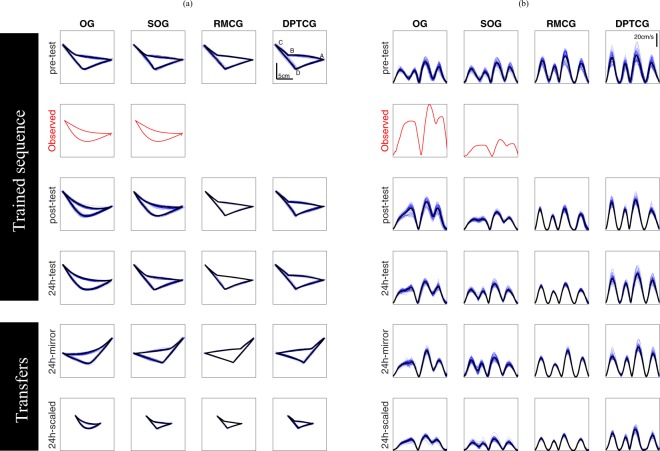
(**a**) All trajectories (blue lines) and mean trajectory (black line) from representative subjects from the four groups (columns), for the five tests (rows). All graphs have the same scale. The x axis corresponds to left-right movements, while the y axis corresponds to forward-back movements. The red trajectories in the second row correspond to the expert’s video recordings shown to the OG and SOG groups. (**b**) Time-normalized tangential velocity profiles (blue lines) and mean tangential velocity (black line) from representative subjects from the four groups, for the five tests. All graphs have the same scale. The x axis is normalized time, while the y axis is the tangential velocity. The red velocity profiles in the second column correspond to those from the videos shown to the OG and SOG groups. Velocity profiles with 4 peaks indicate that the movement can be segmented into four movements between the corresponding points, whereas those with three peaks indicate that movements from A→B and B→C show a large degree of overlap or coarticulation.

Qualitatively different changes in performance evolved following different forms of practice. Movement trajectories (Fig. 1(a)) and tangential velocity profiles (Fig. 1(b)) are shown for the trained layout (pre-training, post-training and 24h, day2 post-training, tests) and the two transfer conditions (mirror and scaled) from representative subjects from the four groups. At the pre-training test, participants of all groups used straight paths (segments) to connect between targets. Each individual segment showed a bell-shaped velocity profile - a characteristic feature of straight point-to-point movements^38^. Following training, movement trajectories connecting A-B-C and C-D-A targets were more curved and longer in the observation groups (OG and SOG) than those generated by participants of the non-observation groups (RMCG and DPTCG) (Fig. 1(a)). Moreover, velocity profiles showed that no complete stops were performed at the points B and D by participants of the observation groups (Fig. 1(b)). Below we present separate analyses of task performance, subdivided to components of movement duration, spatial error and coarticulation measure.

### Movement duration

**Figure 2:**
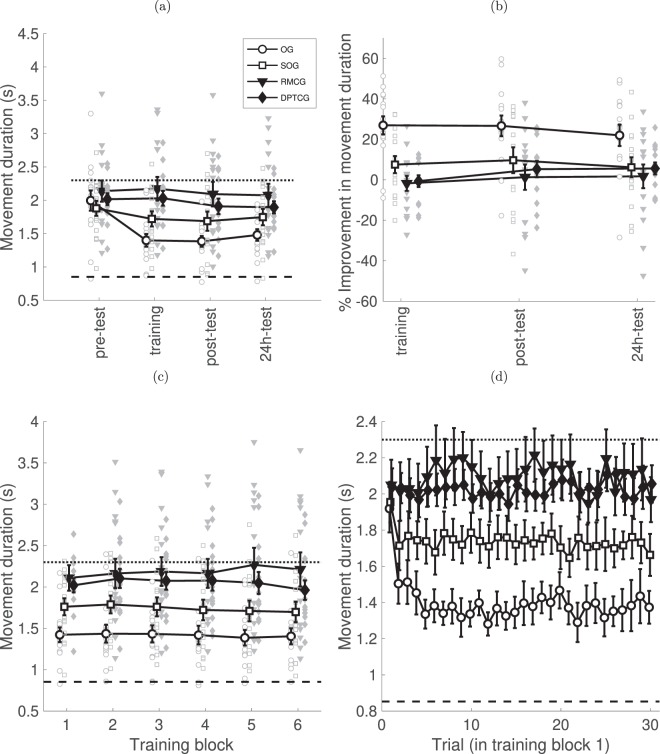
Time-course of changes in movement duration. (**a**) The mean (across subjects) of the median of absolute movement duration per block (in s) and the distribution of individual scores at four time points – pre-training, training, post-training and 24 h post-training. Bars – SE; Dotted line – movement duration of the slowed down expert model (note, that all experimental groups produce faster movements than the slowed model), Dashed line – movement duration of the expert model (note that even participants of the OG do not reach the short movement duration of the expert model). (**b**) Same data as in (**a**) but normalized to the value from the first test session. (**c**) Block-by-block absolute duration during training. The slopes of the movement durations during training were not significantly different from 0. (**d**) Trial-by-trial changes of the movement duration in the first block. Data for all subjects is shown in Supplementary Fig. S1. Bars – SE.

First, to probe the effect of training on movement duration, we compared absolute movement duration before and after training (pre-test, post-test) between groups using a mixed-design ANOVA (Fig. 2(a)). There was no main effect of group (F(3, 55) = 2.511, p = 0.068). While a main effect was found for session (pre-test vs. post-test; F(1, 55) = 14.88, p < 0.001), we focus on the observed interaction between session and group (F(3, 55) = 4.312, p = 0.008). Post-hoc tests showed that only for the observation group (OG) was a significant reduction in time observed, from 2.00 ± 0.15 s to 1.38 ± 0.08 s (t(14) = 4.528, p < 0.001), whereas for all other groups, the change in duration was not statistically significant (all p > 0.1). We performed a similar mixed-design ANOVA on the normalized data, where a main effect was observed for group (F(3, 55) = 4.064, p = 0.011), session (F(1, 55) = 14.806, p < 0.001) and their interaction (F(3, 55) = 4.064, p = 0.011). Again we focus on the interaction, and post-hoc t-tests showed that movement duration was reduced as a result of training in the post-test only for the observation group – Fig. 2(b) (26.7% improvement, t(14) = 5.218, p < 0.001). Analysis of movement duration over the training and post-training time window (training, post-test, 24 h-test) using a mixed-design ANOVA showed that no significant additional improvements took place following training or overnight (i.e., no main effect of session: F(2, 110) = 1.763, p = 0.176 or an interaction: F(6, 110) = 1.452, p = 0.201). A significant main effect was observed, however, for group (F(3, 55) = 6.029, p = 0.001), due to the aforementioned improvement only in the OG group. Thus, the main learning in the observation group occurred during training, no further improvements in movement duration were found (Fig. 2(b)). Post-hoc pair-wise Bonferroni-corrected t-tests showed no difference in the overall improvement between RMCG, DPTCG and SOG, while OG performed better than all other groups (OG vs RMCG: t(28) = 3.89, p = 0.002; vs. DPTCG: t(27) = 3.37 p = 0.008; vs. SOG: t(28) = 2.73 p = 0.048).

Was the training-dependent improvement in movement duration of the observation groups incremental or stepwise? Block-by-block analysis of the performance during training sessions showed that the improvements in movement time occurred already in the first block of the training (see Fig. 2(c)), no further improvements in movement time were observed during the training. This was confirmed using a mixed-design ANOVA, which found a main effect only for group (F(3, 54) = 798.9, p < 0.001), but not for block (F(5, 270) = 0.794, p = 0.554) or the interaction of block and group (F(15, 270) = 0.989, p = 0.467). We also calculated the slopes of the mean movement durations during the 6 training blocks using linear regression, and the t-tests did not find that the slopes of any of the lines were significantly different from zero (all p > 0.3). To further explore this question, we “zoomed in” on the first training block (Fig. 2(d)), where it can be observed that all the improvement (for the observation group) occurred within the first 5 trials. Full results for all subjects are shown in Supplementary Fig. S1. This was confirmed with a mixed-design ANOVA with each 5 trials averaged (e.g. 1–5, 6–10, etc.), which had a main effect for group (F(3, 54) = 7.880, p < 0.001), and an interaction of group and trial (F(15, 270) = 0.026). Focusing on the interaction, we observe that only for the OG is a significant difference observed between the first 5 trials (1.58 ± 0.11 s) and the following 5 trials (1.36 ± 0.08 s; t(14) = 3.873, p = 0.01), whereas no other significant differences are observed between subsequent sets of 5 trials (all p > 0.05).

### Spatial error

**Figure 3:**
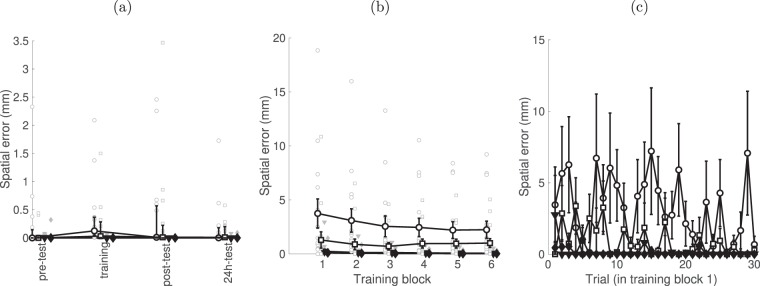
Time-course of changes in accuracy. (**a**) median (across subjects) of the median of spatial error per block and the distribution of individual scores at four time points – pre-training, training, post-training and 24 h post-training. (**b**) Block-by-block spatial error during training. (**c**) Evolution of the spatial error during the first training block (median). Data for all subjects is shown in Supplementary Fig. S2. Bars – IQR.

Altogether, the magnitude of errors (i.e. missing one or more targets) was very low in all groups at all time-points (median less than 0.15 mm for all groups), see Fig. 3. All experimental groups showed very small magnitudes of spatial error at pre-test (medians ± interquartile range: 0.000 ± 0.023 mm), Fig. 3(a). We tested whether the accuracy, quantified by the magnitude of spatial error, changed during the learning process, using non-parametric tests. The Friedman test showed a main effect for spatial error changed across the three tests (pre-test, post-test, 24 h-test, χ^2^(2) = 7.38, p = 0.023) only for the OG group (medians ± interquartile range: pre-test = 0.00 ± 0.19 mm, post-test = 0.01 ± 0.60 mm; 24 h-test: 0.001 ± 0.21 mm; p = 0.023), although post-hoc tests did not find significant differences between the conditions. In particular, we note that the post-hoc Wilcoxon signed rank between the first-day pre-test and 24-h test was not significant (T = 0.267, p = 1.00), suggesting that the trade-off between speed and accuracy during training was transient. Figure 3(b,c) show the change of spatial error during (b) all training blocks, and (c) within the first block. Supplementary Fig. S2 provides data for all subjects for the first block. Due to the lack of significant differences between training and the tests, we did not perform follow-up tests on the training blocks, but include the graphs for completeness.

### Coarticulation (temporal overlap)

**Figure 4:**
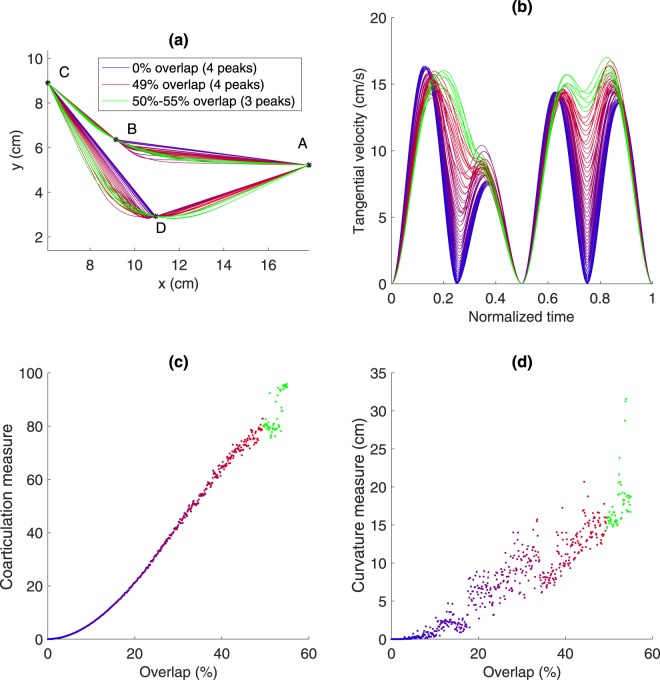
Simulation of movements with varying overlaps and subsequent values for the coarticulation score and the curvature measure. Simulations of 551 overlap values were performed, at equally spaced intervals from 0 overlap to 55% overlap. The movements were generated by assuming the superposition of four minimum jerk submovements^63^, with a given amount of overlap between the first and second, and third and fourth submovements. The location of the first and third intermediate targets was set using non-linear optimization such that the superposition of the trajectories passed through the required points. The code for generating this simulation can be found^64^. In (**a**) and (**b**), every 10^th^ trajectory is shown. (**a**) Trajectories of the simulation at varying levels of submovement overlap, ranging from 0 overlap (in blue) to 55% overlap (in red). (**b**) Normalized tangential velocity profiles, following the same colour scheme. (**c**) Predicted coarticulation measure as a function of movement overlap. (**d**) Predicted curvature measure (in cm) as a function of movement overlap.

As the coarticulation score is a new measure (proposed by the authors), we present in Fig. 4 a simulation of this measure (details are provided in the Figure caption), as a function of movement overlap (see Fig. 4(c)). The measure increases as the amount of overlap increases. In addition, we also used a measure of curvature, based on the notion that increased coarticulation would lead to more curved movements. As the calculation of curvature is problematic (because if the line is perfectly straight, the curvature is infinite), we used as a measure of curvature the mean distance from the straight lines joining the targets (which we call path offset), based on similar measures which have been used previously^39,40^. Analysis of the temporal overlap between motor primitives quantified by three measures (coarticulation score, path offset and number of velocity peaks) is presented below.

**Figure 5:**
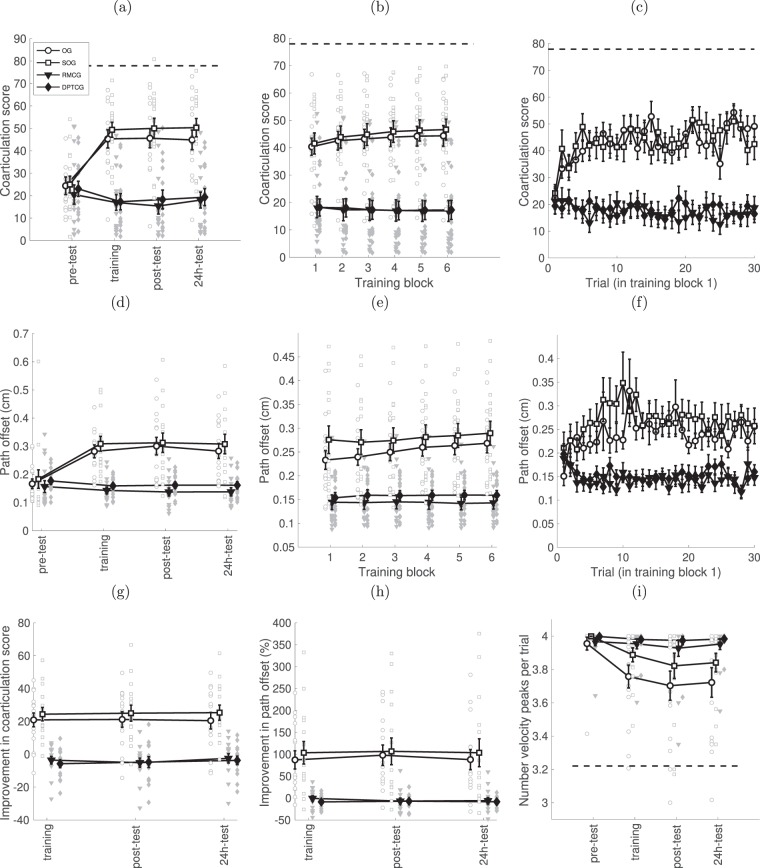
Time-course of changes in coarticulation ability, using three measures. (**a**) Mean (across subjects) of the absolute coarticulation score per block and the distribution of individual scores at four time points – pre-training, training, post-training and 24 h post-training. Dashed line –coarticulation score of the expert model. The grey circles indicate data for all subjects. (**b**) Evolution of the absolute coarticulation score block-by-block during training. The slopes were positive and significantly different from zero only for the OG and SOG groups. (**c**) A further zoom in on the first block. Data of all subjects can be found in Supplementary Fig. S2. (**d**) Mean (across subjects) of the path offset measure, (**e**) Zoom in. on the first block of the path offset measure, (**f**) A further zoom in on the first block, data for all subjects can be found in Supplementary Fig. S3. (**g**) The same data as in (**a**), but normalized to the value from the first test session, and the distribution of individual scores. (**h**) The same data as in (**d**), but normalized to the value from the first test session. (**i**) Mean number of velocity peaks. Dashed line - the number of peaks produced by the expert model. In all graphs, bars – SE.

The magnitude of the coarticulation measure, the major marker of skilled performance in the planar movements sequence task^10^, is shown in Fig. 5(a–c). To probe the effects of different types of training on coarticulation ability, we ran a mixed-design ANOVA comparing absolute coarticulation scores before and after training (pre-test, post-test). A main effect was observed for session (F(1, 55) = 20.178, p < 0.001), group (F(3, 55) = 8.213, p < 0.001) and the interaction of session and group (F(3, 55) = 16.172, p < 0.001). Again, we will focus only on the interaction: post-hoc tests showed that only the observation groups (OG and SOG) showed large, significant improvements following training (OG: increase of 21 ± 4, t(14) = 4.477, p = 0.001; SOG: increase of 25 ± 4; t(14) = 5.112 p < 0.001). The improvement in the coarticulation score of the other two groups with no observation (RMCG and DPTCG) were not significantly different from zero following training, compared to pre-test performance (Fig. 5(g)). A mixed-design ANOVA on the relative improvement with three time-points (training, post-test, 24 h-test) and four groups showed a main effect of group (F(3, 55) = 18.316, p < 0.001), but no main effect for session or interaction (Fig. 5(g)). Post-hoc Bonferroni-corrected pairwise t-tests showed that the mean coarticulation values for OG (21 ± 4) and SOG (25 ± 4) were both better than RMCG (−4 ± 4) and DPTCG (−5 ± 4) (OG vs. RMCG: t(28) = 4.77, p < 0.001; OG vs. DPTCG: t(27) = 4.88, p < 0.001; SOG vs. RMCG: t(28) = 5.55, p < 0.001; OG vs. DPTCG: t(27) = 5.65, p < 0.001). Significant differences were not observed between groups OG and SOG, nor between RMCG and DPTCG (all p > 0.4).

Large improvements in the coarticulation measure were evident already in the first block of the training (see Fig. 5(b)). Moreover, in contrast to the time-course of improvement in movement duration, the coarticulation measure for the two observation groups, following the first grand gain in the first block of training, continued to improve during the training, whereas the other two groups did not. This was confirmed by a mixed-design ANOVA on the four groups and the six blocks, which showed a main effect for group (F(3, 55) = 17.503, p < 0.001), for block (F(5, 275) = 4.206, p < 0.001) and the interaction of block and group (F(5, 275) = 4.412, p < 0.001). Post-hoc one-tailed t-tests on the slopes on a per-subject basis were positive and significantly different from zero only for the observation groups (OG: slope = 0.73 ± 0.37, t(14) = 1.98, p = 0.034; SOG: slope = 0.97 ± 0.32, t(14) = 3.04, p = 0.0044). We note, however, that as with the movement time, most of the gain in the first block occurred during the first few movements of the first block, see Fig. 5(c). A mixed-design ANOVA on the coarticulation measure (on groups of 5 trials) showed a main effect for group (F(3, 54) = 15.365, p < 0.001), for trial (F(5, 270) = 5.421, p < 0.001), and their interaction (F(15, 270) = 4.758, p < 0.001). Focusing on the interaction, we observed significant differences between the coarticulation measure in the first five trials and the subsequent five trials only for the OG group (t(14) = −3.460, p = 0.02), with no further significant differences observed between sets of five trials.

We confirmed these results using a direct correlate of coarticulation ability, - a measure of curvature, namely path offset. A mixed-design ANOVA comparing pre-test and post-test showed a main effect for session (F(1, 55) = 18.190, p < 0.001), group (F(3, 55) = 6.222, p = 0.001) and their interaction (F(3, 55) = 10.277, p < 0.001), Fig. 5(d). Focusing on the interaction, post-hoc tests showed improvement (larger path offset) only for the two observation groups (OG: increase of 0.14 ± 0.03 cm, t(14) = 4.633, p < 0.001; SOG: increase of 0.13 ± 0.04 cm, t(14) = 3.105, p = 0.008). When comparing normalized improvement in path offset over training, post-test and 24 hr test (Fig. 5(h)), a main effect was similarly only observed for group (F(3, 55) = 10.693, p < 0.001). Post-hoc t-tests showed that the two observation groups increased path offset (OG: 91.0 ± 18.3%, SOG: 104.6 ± 18.3%) more than the control groups. A mixed-design ANOVA on the training blocks (Fig. 5(e)) showed a main effect for group (F(3, 55) = 13.753, p < 0.001), block (F(5, 275) = 5.585, p < 0.001) and interaction of group and block (F(15, 275) = 2.335, p = 0.004). Post-hoc one-sided t-tests on the slopes showed a significant improvement only for the OG group (slope: 0.0074 ± 0.0028, t(14) = 2.6782, p = 0.009). Similar to the other measures, the change in path offset also occurred mostly in the first few trials of the first block (see Fig. 5(f)). A mixed-design ANOVA on groups of 5 trials showed a main effect for group (F(3, 54) = 12.060, p < 0.001), and an interaction of group and trials (F(15, 270) = 2.629, p = 0.001). Focusing on the interaction, post-hoc tests showed a significant difference between the first 5 trials and the subsequent five for the SOG group (p = 0.007), and marginally significant difference for the OG group (p = 0.061). The remainder of the differences were not significant.

An additional direct correlate of coarticulation ability is the number of velocity peaks during performance trials (Fig. 5(i)). The mean number of peaks was quantified for the different groups over time. A mixed-design ANOVA showed a main effect of group (F(3, 55) = 4.09, p = 0.011), session (F(5, 275) = 11.370, p < 0.001) and their interaction (F(15, 275) = 1.864, p = 0.027). We focus our presentation of results on the interaction. Specifically, a repeated measures ANOVA performed individually on each group showed a main effect of session only for the OG and SOG groups (OG: F(5, 70) = 5.842, p < 0.001; SOG: F(5, 70) = 4.336, p = 0.002). That is, only for these groups did the mean number of peaks significantly change over the course of two experimental days.

### Transfers

**Figure 6:**
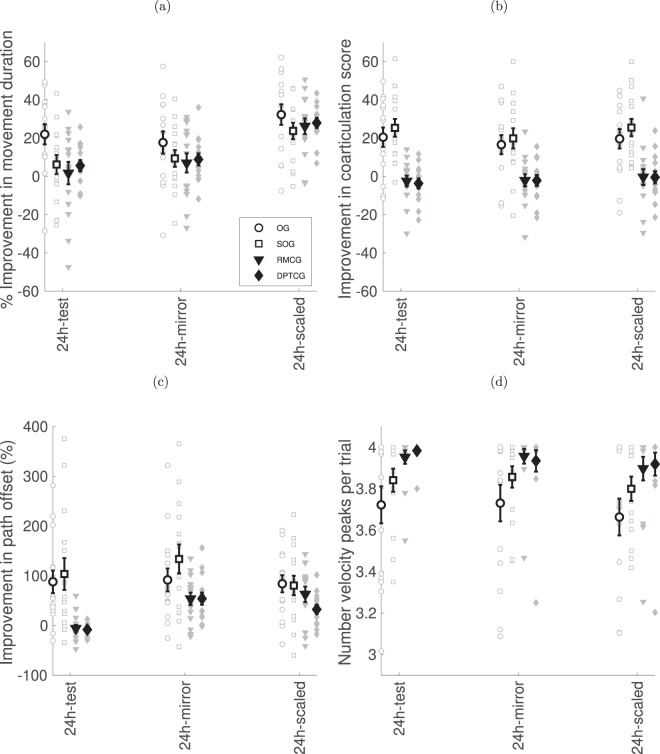
Performance at two transfer conditions (mirror-reversed and scaled layout) in comparison to trained condition performance at the 24 h re-test. (**a**) Relative improvement of the movement duration. (**b**) Relative improvement in the coarticulation score. (**c**) Relative improvement of the path offset measure. (**d**) Mean number of velocity peaks.

To infer which task features were learned in a condition-specific way and which were generalizable across other conditions of performance, at the second experimental day two transfer conditions were afforded – a mirror-reversed movement sequence (test for order specificity) and scaled target layout (test for path-length specificity). The results are shown in Fig. 6.

We compared the improvement in the transfer trials to the test performed at the start of the second day for the trained condition (24 h-test). A significant main effect was observed for transfer type for the relative improvement in movement duration (see Fig. 6(a); F(2, 110) = 150.0, p < 0.001). This was due to the improvement being greater in the Scaled condition (27.5 ± 2.1%) compared to the other two conditions (24 h-test of the trained condition: 8.9 ± 2.5%; t(58) = 13.1, p < 0.001; mirror reverse: 10.7 ± 2.4%, t(58) = 14.2, p < 0.001). No main effect was found for group (F(3, 55) = 1.63, p = 0.19); however, an interaction of group and transfer type was found (F(6, 110) = 3.87, p = 0.02). No group showed a decrease in performance in the Mirror reversed transfer trials compared to the 24-h test, however only the RMCG group showed a very small improvement in the Mirror reverse transfer condition (24 h-test: 1.7 ± 5.9%, 24 h-mirror: 7.0 ± 5.0%, t(14) = 2.59, p = 0.02).

A significant main effect was observed for transfer type for the coarticulation score (see Fig. 6(b); F(2, 110) = 6.426, p = 0.002). This was due to the coarticulation score being higher for the 24 h-test (9.9 ± 2.0; t(58) = 2.35, p = 0.045) and the scaled transfer condition (8.0 ± 2.1; t(58) = 3.18, p = 0.008) compared to the reverse transfer condition (8.0 ± 2.1). As before, a significant main effect was observed for group (F(3, 55) = 10.959, p < 0.001), following the same patterns as previously, i.e. mean coarticulation values for OG (18.9 ± 4.0) and SOG (24.6 ± 4.0) were both better than RMCG (1.7 ± 4.0) and DPTCG (−2.1 ± 4.2) (OG vs. RMCG: t(28) = 3.48, p = 0.004; OG vs. DPTCG: t(27) = 3.62, p = 0.004; SOG vs. RMCG: t(28) = 4.42, p < 0.001; SOG vs. DPTCG: t(27) = 4.59, p < 0.001. The interaction of group and transfer type was not significant (F(6, 110) = 2.068, p = 0.063).

For the path offset measure, Fig. 6(c), a main effect was observed for group (F(3, 55) = 4.778, p = 0.005), reflecting the significantly longer path offsets for the slow observation groups (SOG: 105.9 ± 17.5%) compared to the control groups (RMCG: 36.8 ± 17.5%, t(28) = 2.47, p = 0.04; DPTCG: 26.0 ± 18.1%, t(27) = 2.90, p = 0.02). A main effect was also found for transfer type (F(2, 110) = 18.830, p < 0.001), as post-hoc t-tests showed that the relative path offset for the reverse condition (83.2 ± 10.3%) was greater than for the scaled condition (64.8 ± 8.0%; t(58) = 2.62, p = 0.019), which was greater than for the 24 h-test (44.3 ± 10.2%; t(58) = 27, p = 0.015). The interaction of group and transfer types also showed an interaction (F(6, 110) = 6.693, p < 0.001). Post-hoc tests showed that for the control groups, the mirror condition (RMCG: 53.6 ± 12.3%; DPTCG:53.9 ± 12.2%) and the scaled condition (RMCG: 62.7 ± 15.5%; DPTCG: 32.3 ± 9.0%) showed longer path offsets than for the 24 h-test (RMCG: −5.8 ± 6.9%; DPTCG:−8.3 ± 3.7%), whereas no significant differences were observed for the observation groups between the transfer conditions (RMCG: mirror vs. 24 h: t(14) = 6.37, p =  < 0.001, scaled vs. 24 h: t(14) = 4.97, p < 0.001; DPTCG: mirror vs. 24 h: t(13) = 5.67, p < 0.001, scaled vs. 24 h: t(13) = 4.64, p < 0.001).

For the number of peaks (see Fig. 6(d)), there was a main effect of group (F(3, 55) = 3.783, p = 0.015), due to the OG having a lower number of peaks than both control groups (OG: 3.71 ± 0.06, RMCG: 3.94 ± 0.06, t(28) = 2.46, p = 0.037; DPTCG: 3.95 ± 0.06, t(27) = 2.47, p = 0.03). There was also a main effect of session (F(2, 110) = 5.222, p = 0.007), as the number of peaks for the Scaled condition (3.82 ± 0.03) was slightly lower than for the trained (3.87 ± 0.03, t(58) = 2.78, p = 0.026) and Mirror (3.87 ± 0.03, t(58) = 3.09, p = 0.012) conditions. The interaction was not significant.

In terms of spatial error, a Friedman test showed a main effect for transfer type (χ^2^(2) = 59.3, p < 0.001). Post-hoc Wilcoxon signed ranked tests showed that the spatial error was greater for both the Mirror reverse condition (median = 1.83 mm; T = 1.26, p < 0.001) and the Scaled condition (median = 1.47 mm; T = 1.18, p < 0.001) compared to the trained condition (median = 0.00). Kruskal-Wallis tests showed that differences were observed between groups for 24 h-test (H(3) = 12.43, p = 0.006) and the Mirror reverse condition (H(3) = 21.24, p < 0.001) but not for the Scaled condition (H(3) = 3.94, p = 0.268). Post-hoc Mann-Whitney U tests showed significantly lower spatial errors for the Mirror reverse condition for both RMCG (0.23 ± 0.60 mm) and DPTCG (0.67 ± 0.86 mm) compared to both OG (2.42 ± 4.22 mm) and SOG groups (2.00 ± 2.94 mm), (RMCG vs. OG: T = 22.50, p = 0.002; RMCG vs. SOG: T = 21.6, p = 0.004; DPTCG vs. OG: T = 19.3, p = 0.015, DPTCG vs. SOG: T = 18.4, p = 0.024).

## Discussion

Learning of a novel sequential motor skill implies acquiring fluency in the execution without compromising accuracy. In many cases, including sequences of planar hand-writing movements, skilled performance is not based on faster execution of the already familiar or simple basic movements (primitives), but on the evolution of new motor primitives, gradually emerging through repetitive practice of the task from temporal fusion of basic motor primitives^10,41–43^. A novel movement strategy may lead to a longer path but affords smoother and faster performance engaging a smaller number of motor primitives. For the task employed in the current study, previous studies have shown that extensive multi-session training is needed to achieve fluent execution based on novel motor primitives^10^. Here, we tested whether observation of the movement pattern of the expert model (without observation of the movement trace that would provide an explicit solution), intermittent with physical practice may facilitate the acquisition of the co-articulated motor planning strategy in task-naïve participants. Two observation conditions were afforded in different groups of subjects – in one the expert’s performance was shown at its original speed, while in the second, a slowed expert’s performance was shown, to allow task-naïve participants to observe co-articulated performance at a speed close to their own. In two control training conditions, double physical practice or observation of random dots movement intermittent with physical practice were afforded. Observation groups showed robust gains in coarticulation ability, evident in an increase in the coarticulation score (temporal fusion of motor primitives), increase in the path offset (mean distance from the straight lines joining the targets) and a reduction in the number of velocity peaks. This improvement happened very fast during practice: robust gains in performance evolved already within the first 5–10 trials of the first intermittent block of the training session, however, additional slow improvements in coarticulation continued to evolve through five additional practice blocks. No offline delayed gains in performance were observed over the night’s sleep period^34,44^, in line with previous studies^10,18^. The fast within session improvements in coarticulation ability are likely to be subserved by priming effects^36,45^. The stabilisation of the coarticulated performance pattern during the retest on the 24 hr session without observation suggests engagement of between session consolidation processes^4,46^. This is further supported by the time-course of changes in the accuracy, characterised by initial within-session decreased accuracy, followed by a return to baseline high accuracy at 24-hour testing. Altogether, this suggests that the presumably primed knowledge of coarticulated movement strategy was fully consolidated and integrated into the long-term representation of the task within a 24 hr time-window.

The speed of the expert’s model presentation, original or slowed down, did not affect the magnitude of the coarticulation evolved. In contrast, learning of movement duration was affected by the model’s speed, with the OG achieving shorter movement durations during training and maintaining it during immediate and 24 h post-tests. This result suggests that coarticulation and movement duration are dissociable processes, as coarticulation ability does not necessarily drive the movement speed. While increased coarticulation assumedly allows the task to be completed faster because less time is spent at slower velocities, the differences between the movement duration of the OG and SOG groups suggest that the improvement in coarticulation does not lead to an automatic corresponding improvement in reducing movement duration. Both the OG and the SOG, however, did not achieve the level of coarticulation or movement duration of the observed model during training or re-tests. We conjecture that while the observation of the expert’s action effectively modified the movement strategy of the observers to fit the visually captured representations of sensorimotor transformations to their own abilities^47,48^, the limited amount of physical practice afforded in a single session restricted the extent to which this strategy could be integrated into the long-term representation of the task.

In line with previous studies^10,18^, our results show that a single practice session without observation of a model did not lead to the coarticulated movement pattern, even if the number of physical repetitions of the task was doubled. Observation of random dots motion did not affect the initial non-coarticulating motor strategy either. As found in previous studies of this task, we assume that several days of training would have led a coarticulated movement pattern.

Only in the observation groups was a significant increase in the magnitude of errors observed immediately after training. This effect was transient: at 24 hours post-training re-test, the novel movement pattern was well-retained in the long-term memory, accompanied by improvement in the magnitude of errors to practically zero level. Thus, practically, by the 24 h post-training, no speed-accuracy trade-off was found for the observation training conditions, suggesting that consolidation of knowledge from observation does not impose long-term accuracy costs.

The strategic outcomes of observation training were effectively transferable to the untrained conditions, requiring performing the task in a reversed movement direction across targets (spatial layout of the targets remained fixed) or when target distances were downscaled (movement direction remained fixed). No group showed a decrease in performance with transfer, while one group showed a very small improvement in movement duration. In both transfer conditions tested at Day 2, the curved movement trajectories were spontaneously generated with a level of coarticulation similar to that achieved in the trained condition for the OG and the SOG. In contrast, no coarticulation was found in transfer tests of the non-observation groups. Movement duration was generalizable as well. In the scaled test, as expected, movement duration showed significant improvements in all groups. This was due to the fact that target size was kept constant (was not down scaled) while the distance between targets decreased. Thus, according to Fitts’ law^49^ the time to complete a movement sequence decreased (Fig. 6(a)). Note, that coarticulation scores are similar across transfer tests, while movement times are significantly different (Fig. 6(a,b)). Thus, Fitts’ law does not predict differences in coarticulation ability, as evident from the dissociation between the observation and the non-observation groups in coarticulation scores and mean velocity peaks, suggesting that improvement in movement duration and improvement in temporal organization of motor behaviour are independent processes.

Earlier studies of a similar hand drawing task suggested that the new motor primitive evolves only if the motor system has reached optimal performance in the global planning of two segments^10^. Our results show that in some training conditions, providing observation of expert model motor performance pattern, this notion does not hold. The role of vision in the evolution of coarticulation ability was recognized in a series of experiments when the task was practiced in darkness^18^. It was conjectured that that self-observation of the hand movement path is crucial for evolution of coarticulation through the learning process^18^. Here we showed that if the participant is afforded a visual observation of the expert’s model hand coaligned with the location of their own hand producing the movements, the course of motor learning is condensed, presumably through a priming mechanism subserved by the action-observation network (AON)^48,50^. Embodiment of the observed action of the model is thought to be promoted by viewpoint and handedness^51^, allowing direct transformation of the observed movements into the observer’s internal coordinate system of motor action^52–54^. Our results are in line with the findings from arm reaching experiments, where viewing another person performing a reaching task with obstacle avoidance^45^ primed the following actions of the observer, but only if the obstacle was placed within the action (peripersonal) space of the observer.

In this study, we defined a new measure of coarticulation, namely the ratio of the heights of the troughs to the heights of the peaks, in the tangential velocity profile. Using a simulation of the superposition of minimum jerk trajectories (Fig. 4), we showed that this measure captures well the amount of overlap between submovements (i.e. coarticulation). Moreover, when directly compared to a purely spatial measure (path offset), it is better able to differentiate between trajectories with a relatively small amounts of overlap. Additionally, we note that in the transfer conditions, the path offset showed greater path offset for the control groups, while the coarticulation measure did not. The increase in path offset was likely spurious, and caused by greater inaccuracies rather than actual coarticulation (as seen from the coarticulation score). The coarticulation score may prove useful in further studies, as a way of tracking how subjects overlap their execution of movements, without requiring explicit decomposition of the movement into submovements, a procedure which may lead to the identification of spurious movements^55,56^.

Observation training is increasingly suggested as a therapeutic approach in motor rehabilitation^57^. In fact, ecological and rehabilitation settings of drawing and writing often include observation practice, either of path or of movements, or both^22^. Thus, two important questions should be raised, before including broad implementation of observation learning of complex planar hand actions: Can practice by observation induce implicit optimization of movement strategy? Does practice by observation result in procedural knowledge that is similar to the knowledge (skill) created by physical practice? Our results show that learning of a qualitatively different motor plan can be facilitated to a large degree by observation of such strategy from an expert model intermittent with physical practice.

A limitation of this study is that it does not allow us to resolve directly the question whether the step-like change in the motor strategy is an explicit insight or an implicit process, e.g., perceptual priming. Robust improvements in the coarticulation measure were evident already in the first block of the training (see Fig. 5(c)), showing a step-like improvement in both in the domain of temporal organization and in the movement duration (Fig. 2). There are no direct indications whether this change of the motor pattern based on a novel, curved, trajectory instead of concatenation of consecutive segments, happened implicitly or explicitly during training. However, based on the fact that velocity profiles during training and at the post-training tests correspond to a four-segment movement and not a three-segment movement (as in the expert model), we conclude that the improvement occurred implicitly. If the participants would explicitly change their movement strategy from a four-segment to a three-segment movement, we would expect them to use B as a via-point^38^, which would lead to single-peaked velocity profile between points A and C (see Figs 1 and 8), but this was not observed. This conclusion is further supported by the fact that the coarticulation score continued to improve incrementally through the course of training (Fig. 5(b)) and the number of velocity peaks continued to decrease after the end of training (Fig. 5(i)). Thus, the participants experienced a large step-like improvement in motor strategy, though not ideal, followed by further incremental improvements. On the other hand, the fact that the coarticulated performance fully generalized to transfer conditions, in which the motor plan had to be internally generated, is ambiguous. It may be both due to explicit or implicit task representations. An additional limitation of this study is that only right-handers were included, and only a single task was used. As subjects were tested over only two days, we did not test whether the relative improvement in performance by the observation groups compared to the other groups remains after multiple days of training, or whether the performance of the other groups catches up.

Altogether, our results, in line with^34,58,59^, suggest that although practice by observation of an expert’s movements is a very effective learning experience, - it saves extensive training to reach the concept of co-articulation of movement components, the resultant procedural knowledge is qualitatively different from the knowledge (skill) created by multi-session physical practice.

## Methods

### Participants

60 participants (15 in each group) took part in the study (37 females), recruited from the student population at Tel Aviv University. The sample size was based on the effects observed in Sosnik *et al*.^10^, where in terms of movement time, the group that performed coarticulation improved by 57.1%, whereas the group that did not perform coarticulation improved by 39.7%. As we expect the observation groups will perform coarticulation, and the control groups will not, we expect to observe similar differences in improvement in movement time. Based on a standard deviation in each group of 12.5%, and a power of 0.95, we require 15 subjects in each group, based on a mixed-design ANOVA design^60^. Ethical approval was received from the Tel Aviv University Institutional Review Board, and participants signed an informed consent form before beginning the experiment. The study was performed in accordance with the relevant guidelines and regulations. Participants were paid for their participation (70 New Israel Shekels, approximately $15).

### Apparatus

The hand movements were recorded at 160 Hz using a stylus on a tablet computer (Samsung XE700T1C, 11.6” screen, 1920 × 1080 resolution) running Microsoft Windows 8, using the Repeated Measures Matlab-based software^61^. The tablet computer was placed flat on a table at a comfortable distance.

### Training of the “expert” and generation of the model stimuli

In order to generate expert performance to show for the observation trials, a single subject performed the training over 10 sessions on different days (6 blocks of 30 movements in each session). Following the 10 sessions, her movement was filmed using a video camera from the view point of the participant (see Fig. 6). The stimulus for the observation groups included presentation of a single video recording trial of performance, shown on the tablet screen. The size of the video was rescaled such that the targets were the same size and located with exactly the same layout as in the physical training trials. Thus, the movements of the hand holding a stylus were observed exactly as they would appear if the task would have been performed by the participant him or herself. No variability was added to the observation trials (i.e. the same observation video was played in all observation trials).

### Study Protocol

**Figure 7:**
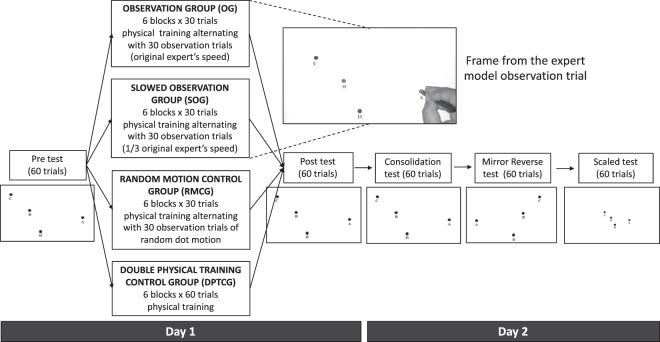
Experimental protocol. Day 1 included: Pre-test of performance, Training and Post-test of performance, Day 2 included: Consolidation performance test and two transfer tests – Mirror Reverse order of targets and Scaled (reduced distance between targets) tests.

The experiment protocol is laid out in Fig. 7. The participants were randomly assigned to one of four groups, with each group receiving a different type of training. The layout of the stimuli was based on stimuli used in a previous study^10^. The participants were instructed to place the stylus on the dot next to the letter “A”, and then, as quickly and accurately as possible, pass through the other dots in alphabetical order and return to “A” (i.e. A→B→C→D→A), and wait there until the dots disappeared (which occurred when stopped on A for 500 ms). The diameter of the dots was 5.4 mm. Performance feedback (path or accuracy of passing through the dots) was not provided. During the first experimental session (day1), all subjects started with a test session, with 60 trials, followed by a training and a post-training re-test. A 1500 ms break was given between trials. All groups had six blocks of training with 60 trials in each block, with the training for each group as follows (where a training trial is the same as the trials in the test block):

1. *Observation group (OG) –* each block consisted of one trial training, alternating with one trial of video playback of an expert performing the task (30 training trials, 30 observation trials).
2. *Slowed observation group (SOG)* – each block consisted of one trial training, alternating with one trial of video playback of an expert performing the task at 1/3 speed (30 training trials, 30 observation trials)
3. *Random motion control group (RMCG)* – each block consisted of one trial training, alternating with one trial of random dot motion (30 training trials, 30 observation trials).
4. *Double physical training control group (DPTCG)* – each block consisted of 60 training trials.

On the next session (day 2), 24 hours following first session, three performance tests (60 trials each) were afforded to participants: (i) Trained consolidation test - trained spatial layout and order of targets, (ii) Mirror transfer test – mirror reversed (left-right) spatial layout requiring a mirror-reversed movement order, (iii) Scaled transfer test - spatial layout that was scaled down by 30% from the original, but the original target sizes and order were maintained (see Fig. 7).

### Analysis

Trials were removed from the analysis if the stylus position was not recorded continuously from the start to the end point (3.3% of trials). The raw data were filtered using smoothing splines, with knots every 6 samples. For each trial, movement onset was defined as the last time (before the first tangential velocity peak greater than 35% of peak tangential velocity) that the tangential velocity was less than 5% of the peak tangential velocity. The end of the movement was defined as the last time the tangential velocity was greater than 5% of the peak tangential velocity. This allowed calculation of the movement time, defined as the time between movement onset and the end of the movement. In order to display plots of mean trajectories, we first registered (aligned) the data^62^. We performed the analysis on both the absolute measures and the normalized to the pre-training performance data sets. The normalization (percent improvement) was performed by subtracting the movement duration from the baseline value (from the pre-test block), then dividing by the baseline value and multiplying by 100. Spatial error was quantified by measuring the closest distance of the subject’s movements from the edge of each of the specified points, and then summing them. If the subject successfully passed through all the points (i.e., was within the borders of the targets), the spatial error was defined as zero.

**Figure 8:**
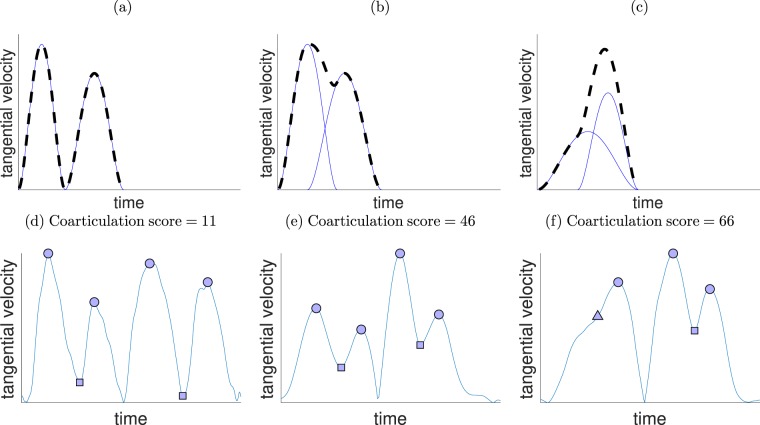
Explanation of coarticulation and computation of the coarticulation score. (**a**–**c**) Show three examples of overlap of submovements (in blue). The observed movement is the sum of these submovements (dashed black line). When there is some overlap (**b**), the height of the trough increases. With sufficient overlap (**c**), the trough disappears, although we still observe an inflection point (marked as a triangle). (**d**–**f**) show three representative performance trials from different stages of learning. The coarticulation score was defined as the ratio of the mean value of the tangential velocity troughs (marked by squares) to the mean value of the tangential velocity peaks (marked by circles). When a peak/trough “disappears” as in (**f**), the peak and trough are replaced in the calculation with the inflection point. In example (**d**), showing a single pre-training trial, there is very little coarticulation (score = 11), whereas in example (**e**), showing a single training trial, there is a much higher amount of coarticulation (score = 46). Example (**f**) shows a single post-training trial featuring only 3 peaks, i.e., greater coarticulation (score = 66).

Coarticulation is defined as an overlap of two movement primitives (or submovements) – see Fig. 8(a–c). We assumed that when there is an overlap of submovements, we will observe a superposition of the submovements velocity profiles^63^. This overlap results in the troughs in the tangential velocity profile being greater than zero (Fig. 8(b)). With sufficient overlap (Fig. 8(c)), the trough disappears, and a new motor primitive emerges. Thus, we defined a coarticulation score as the ratio of the mean of the first and third trough heights to the mean of the peak heights (see Fig. 8), multiplied by 100. We considered the heights of the 4 largest peaks, with the lowest points between them defined as the troughs. We did not include the second trough which was typically zero (because of the change in direction required by the task). When there were only 3 peaks (due to coarticulation), we used the inflection point (that replaced the peak and trough) for both the missing peak and trough. A similar procedure was performed when there were only two peaks. Normalization of coarticulation scores was performed by subtracting the baseline value. In this case, we did not divide by the baseline value, because many baseline values were close to zero, thus dividing by these values would have produced very large numbers. A low coarticulation score (close to zero) indicates that the subject did not coarticulate (i.e. made 4 distinct point-to-point movements), a higher score (closer to 100) indicates greater coarticulation (i.e. did not stop at the intermediate targets and smoothly changed direction).

To measure the accuracy, we calculated the spatial error, which we defined as the mean (over trials) of the summed distance from each target to the closest point on the path of the stylus. If the stylus intersected the target, the distance was set to zero for that target.

### Statistics

As we expect many zero values for the spatial error and thus the measure cannot be normally distributed, we used non-parametric statistics for analysis of this quantity. Parametric statistics were used for the other quantities.

We tested the effect of training by using mixed-design ANOVAs on the four groups, comparing the pre-test and post-test movement times, coarticulation scores, and path offset. We compared the time-course of learning across the groups by performing a mixed-design ANOVA on normalized movement time, relative improvement in coarticulation, relative improvement in path length, and number of tangential velocity peaks, with between-subjects factor of group, and within-subject factor of session (training, post-test, 24 h-test). We compared the effect of learning during the 6 training blocks by performing a mixed-design ANOVA on the 6 training blocks, followed up with linear regression when significant effects were found to test whether the slope is different from zero (using t-tests). We compared the spatial error across training using a Friedman test over the three sessions (pre-test, post-test and 24 h-test).

Transfer was similarly tested using a mixed-design ANOVA on normalized movement time, relative improvement in coarticulation, relative improvement in path offset, and number of tangential velocity peaks, with between-subjects factor of group, and within-subject factor of session (24 h-test, mirror- test and scaled-test). A Friedman test was used for the spatial error.

For the mixed-design ANOVAs, when an interaction of group and session was observed, we focused on the interaction in our presentation of the results, as our primary interest is in how the groups differ as a result of training or during transfer.

Significant effects were followed up with post-hoc, Bonferroni corrected t-tests (for parametric data) or Wilcoxon sign-rank tests (for non-parametric data).

## Supplementary information

Supplementary information

## Data Availability

The datasets analysed during the current study are available from the corresponding author on reasonable request.
