# Supplementary material for: Observation of an expert model induces a skilled movement coordination pattern in a single session of intermittent practice
Source: Sci Rep. 2019 Mar 14;9:4609. doi: 10.1038/s41598-019-40924-9 (PMC6418165; doi:10.1038/s41598-019-40924-9)
Supplement: Supplementary file 1 — Supplementary information [file 41598_2019_40924_MOESM1_ESM.docx]

**Observation of an expert model induces a skilled movement coordination pattern in a single session of intermittent practice**

Jason Friedman and Maria Korman

**Supplementary information**

Supplementary Figure S1. Trial-by-trial presentation of the movement duration for the first training session for the four groups. The gray shapes indicate data for all subjects. Bars – mean and standard error. Dotted line – movement duration of the slowed down expert model. Dashed line – movement duration of the expert model

Supplementary Figure S2. Trial-by-trial presentation of the spatial error for the first training session for the four groups. The gray shapes indicate data for all subjects. Bars – median and inter-quartile range.

Supplementary Figure S3. Trial-by-trial presentation of the coarticulation measure for the first training session for the four groups. The gray shapes indicate data for all subjects. Bars – mean and standard error.

Supplementary Figure S4. Trial-by-trial presentation of the path offset measures for the first training session for the four groups. The gray shapes indicate data for all subjects. Bars – mean and standard error.
